# Affective cues in busy minds: emojis, affect infusion, and brand evaluation

**DOI:** 10.3389/fpsyg.2026.1853413

**Published:** 2026-08-24

**Authors:** Gunwoo Yoon

**Affiliations:** Department of Marketing and Entrepreneurship, David W. Wilson College of Business, University of Northern Iowa, Cedar Falls, IA, United States

**Keywords:** cognitive busyness, emojis, multitasking, social media marketing, the Affect Infusion Model

## Abstract

Emojis are ubiquitous in digital communication, and brands widely integrate them into social media marketing. Drawing on research on cognitive busyness, multitasking, and the Affect Infusion Model, we propose that emojis function as context-dependent affective cues rather than uniformly processed signals. Across two studies, we examine how cognitive busyness shapes their impact on brand evaluations. Study 1 shows that under cognitive busyness, emojis serve as salient affective shortcuts, increasing reliance on heuristic processing and leading people to use emoji-elicited emotions as proxies for brand attitudes. Study 2 further demonstrates that emojis enhance ad liking, but this effect is contingent on perceived selling intent. When cognitively busy, people rely on immediate affective responses, increasing positive evaluations; when not busy, they engage in more deliberate processing, interpret emojis as persuasive cues, and attenuate affect infusion. These findings highlight the boundary conditions and underlying mechanisms of emoji effectiveness in social media marketing.

## 1 Introduction

Emojis are everywhere, largely due to the widespread adoption of digital devices and social media, which has accelerated the use of this intuitive, image-based language (Ge and Gretzel, 2018). Since their emergence, emojis have been recognized as an effective tool for conveying ideas and messages. A substantial body of research indicates that these graphic representations of facial expressions offer multiple communicative advantages. Specifically, emojis can (1) convey emotional states, (2) clarify communicative intent, (3) facilitate the initiation and maintenance of social interactions, and (4) attenuate impersonality and excessive formality, thereby fostering interpersonal relationships (Das et al., 2019; Derks et al., 2008; Glikson et al., 2018; Lo, 2008; Rodrigues et al., 2017). Furthermore, relative to plain text, emojis are often more engaging and nuanced, enhancing message interpretation by functioning as contextualization cues (Li et al., 2019).

Beyond their expressive function in everyday text messaging and social media posting, emojis have also become standard components and pervasive tools in marketing communications. Empirical evidence indicates that these paralinguistic cues are among the most frequently used elements in brand posts on social media, underscoring their role in formatting, emphasis, and tone within brand messaging (Lang, 2025; Li et al., 2019). More recent industry reports further suggest that emojis help capture attention, humanize brand voice, and convey emotional nuance in brand communication, thereby enhancing engagement metrics on platforms such as Instagram, X, and Facebook (Brandwatch, 2023; Gaillot and Amarotti, 2025). Consumer-focused analytics also support these marketing applications, indicating that a substantial proportion of consumers develop emotional connections with brands through emoji usage, while also showing that inappropriate or insensitive emoji choices can negatively affect brand perceptions and even lead to disengagement (Sensi, 2024).

Taken together, the benefits highlighted in the aforementioned industry reports and scholarly studies suggest substantial demand for emojis in brand communications. Emojis appear to have shifted from being optional stylistic elements to normalized components of digital and social media marketing practice.

However, marketers today do not simply adopt emojis as decorative or communicative elements; rather, emojis have evolved from “cute additions” to strategic non-verbal anchors in brand communication. For instance, Domino’s emoji-based ordering system illustrates how emojis can function as behavioral shortcuts, translating consumer intent into immediate action (Danesi, 2017). Similarly, Chevrolet emoji-only messaging demonstrates how emojis act as cultural signals, enabling brands to convey digital fluency and align with younger audiences despite potential ambiguity (Evans, 2017). The World Wide Fund for Nature’s #EndangeredEmoji campaign further highlights how emojis can serve as engagement mechanisms, transforming symbolic expression into tangible outcomes such as donations and social participation (World Wide Fund for Nature [WWF], 2015).

Yet, this raises an important empirical question of whether the mere inclusion of emojis in companies’ social media posts always enhances brand communication and produces positive downstream effects. We argue that, even with AI-driven emotional optimization systems (i.e., use machines to generate, recommend, or optimize emojis), their effectiveness remains contingent on context, execution, and consumer awareness.

## 2 Theoretical background

Consumers typically browse social media on smartphones and other digital devices in fragmented, attention-constrained environments, where multiple streams of information compete simultaneously (Ophir et al., 2009). Rather than engaging in fully focused processing, consumers often scroll through feeds in short bursts, rapidly shifting attention across posts, advertisements, notifications, and embedded media. At the same time, they frequently engage in multitasking activities such as listening to music, messaging, or switching between apps while consuming social content.

Importantly, such cognitive load and multitasking can produce cognitive busyness, which constrains consumers’ ability to engage in detailed perceptual processing of marketing stimuli, shifting attention away from fine-grained features toward more heuristic cues (Duff et al., 2014; Yoon et al., 2021). Prior literature has shown that as consumers increasingly encounter brand messages while simultaneously engaging in other tasks, advertising effectiveness depends on cues that can persist under reduced attentional capacity (Segijn et al., 2016). This suggests that, as attention is divided, consumers experience conditions analogous to cognitive load, in which limited processing capacity reduces the ability to engage in deep, systematic processing. Consequently, consumers rely more heavily on salient, easily processed elements (e.g., emojis and other prominent symbols) to interpret and respond to content.

If this reasoning holds, we hypothesize that cognitive busyness limits elaborative processing, creating conditions under which consumers are less likely to carefully analyze textual or informational content. Instead, they are more likely to attend to perceptually salient and affectively charged cues–conditions under which emojis may exert a particularly strong influence.

We also base our predictions on the Affect Infusion Model (AIM, Forgas, 1995), which proposes that affect influences judgments to varying degrees depending on the cognitive processing strategy employed. AIM predicts that cognitive capacity constraints increase reliance on affective information. In particular, when people are cognitively busy or distracted, they are less able to engage in systematic analysis and are therefore more likely to use affect as a heuristic for judgment (Forgas, 2008). Under such conditions, incidental affective cues embedded in stimuli are more likely to be infused into evaluative judgments.

Forgas (1998) further extends this argument by demonstrating that affect shapes cognition and strategy selection more strongly when people face complex or demanding tasks. Although originally examined in negotiation contexts, these findings generalize to consumer decision-making, where limited cognitive resources heighten sensitivity to affective signals.

Applied to social media marketing, this suggests that emojis–by conveying emotional meaning quickly, efficiently, and non-verbally–can infuse affective meaning into otherwise informational or promotional text. It further implies that such paralinguistic cues are particularly influential when consumers are cognitively busy, as they tend to elicit affective responses, reduce cognitive effort, and facilitate faster message processing with minimal mental resources.

Integrating insights from cognitive busyness (Lalwani, 2009; Pontari and Schlenker, 2000), multitasking research (Duff et al., 2014; Yoon et al., 2021), and the Affect Infusion Model (AIM; Forgas, 1995, 2008), we here propose that emojis are not uniformly processed but instead function as context-dependent affective tools in social media marketing. We hypothesize that, under conditions of cognitive busyness, emojis operate as efficient emotional heuristics, increasing the likelihood of affect infusion in brand evaluations.

Specifically, when consumers are cognitively busy (i.e., engaged in multiple tasks simultaneously), the processing environment closely aligns with AIM’s conditions for heightened affect infusion. As a result, consumers are less likely to fully infer the sender’s persuasive intent and instead rely on visually salient cues, forming impressions of the post and the brand through an affect-as-information mechanism. In contrast, when consumers are not cognitively busy, they are more reflective and more likely to engage in elaborative processing of social media content. In such cases, consumers are more likely to recognize the sender’s persuasive intent, and emojis are interpreted in conjunction with textual content. Consequently, affect infusion is expected to be attenuated due to increased analytical scrutiny. To test these hypotheses, we conduct two experiments (Studies 1 and 2) and find consistent empirical support for our theorizing.

## 3 Study 1

As noted by Hotz-Behofsits et al. (2025) and Ge and Gretzel (2018), emojis are increasingly used to express persuasive ideas as rhetorical cues or dynamic vocabularies. In contemporary technocultures, these pictorial meaning-making tools enable brands to interact with and engage consumers more effectively. However, a key question remains: Does emoji use always help brands communicate more effectively with consumers and enhance persuasion? What happens when consumers encounter brand emojis (e.g., in social media or advertising) while simultaneously engaging in other tasks and therefore experiencing cognitive busyness (e.g., reading other content on social media, conversing with others, or watching television)? In Study 1, building on prior research on emoji rhetoric, multitasking, and cognitive busyness, we test the hypothesis that varying levels of cognitive busyness (i.e., cognitive load) moderate the effect of emojis on brand outcomes.

### 3.1 Methods

*A priori* power analysis was performed with G*Power (Faul et al., 2007), and it showed a requirement of at least 125 participants to detect a medium-sized effect at 80% power. Three hundred and sixty participants were recruited from Amazon Mturk (172 females, M_*age*_ = 40.70, SD_*age*_ = 12.57). Study 1 employed a 2 (cognitive busyness: not busy vs. busy) × 2 (emoji: yes vs. no) between-subjects design. All participants were randomly assigned to one of the experimental conditions and informed that they would receive several promotional tweets from a brand. Their task was to review and evaluate these tweets, while those in the cognitive busyness condition simultaneously completed an ostensibly unrelated learning task. No participant was excluded from the data analysis.^1^

#### 3.1.1 Cognitive busyness manipulation

To manipulate cognitive busyness, we adopted a well-established dual-task paradigm (see Gilbert et al., 1988), in which participants perform a primary and a secondary task simultaneously, thereby dividing their cognitive resources. Specifically, participants in the non-cognitively busy condition were instructed that they would watch a commercial presented on the screen (i.e., a 60-s television advertisement for American Express, “Hybrid Life”). In the cognitively busy condition, participants were informed that, in addition to watching the commercial, they would simultaneously complete a secondary task requiring them to count the number of personal pronouns used by the spokesperson in the advertisement. Participants in this condition were also told that they would later answer a question regarding the spokesperson’s use of personal pronouns. All participants completed a manipulation check assessing perceived cognitive busyness, indicating the extent to which they felt they were performing multiple tasks and experiencing reduced attentional resources during the study (a single 7-point item ranging from 1 = Not at all to 7 = Very much).

#### 3.1.2 Emoji manipulation

To ensure consistency with the cover story, participants in the control condition (i.e., no emoji) were presented with a series of promotional tweets from American Express (drawn from the official American Express X account). Each original tweet contained a brief promotional message but did not include any emojis. In the emoji condition, participants were presented with the same tweets, supplemented with graphical emojis. The emojis were selected using representative items from the Lisbon Emoji and Emoticon Database (LEED; Rodrigues et al., 2018). Specifically, we selected emojis that were rated as clearly affective, highly familiar, and minimally ambiguous (e.g., winking, smiling, and kissing face emojis).

#### 3.1.3 Procedure and measure

After completing all experimental manipulations, participants were asked to complete a series of questionnaire items. The primary outcome measure was attitude toward the brand’s social media activity. Consistent with prior consumer research, attitude was measured using adapted semantic differential scales anchored by bipolar adjective pairs (MacKenzie and Lutz, 1989; Spears and Singh, 2004). Specifically, participants reported their evaluations using three 7-point semantic differential items: unpleasant–pleasant, bad–good, and unfavorable–favorable (Cronbach’s α = 0.91). Participants also completed demographic measures and were subsequently debriefed.

### 3.2 Results

#### 3.2.1 Manipulation checks

Participants assigned to the cognitively busy condition (*M* = 4.37, SD = 2.02) reported that they were engaging in multiple tasks and experienced reduced available cognitive resources during the study compared to those in the non-cognitively busy condition (*M* = 3.43, SD = 2.05), *t* (358) = 4.39, *p* < 0.01, *d* = 0.46, 95% CI = [0.25, 0.67]. Thus, our manipulation of cognitive busyness was successful.

#### 3.2.2 Hypothesis testing

**FIGURE 1:**
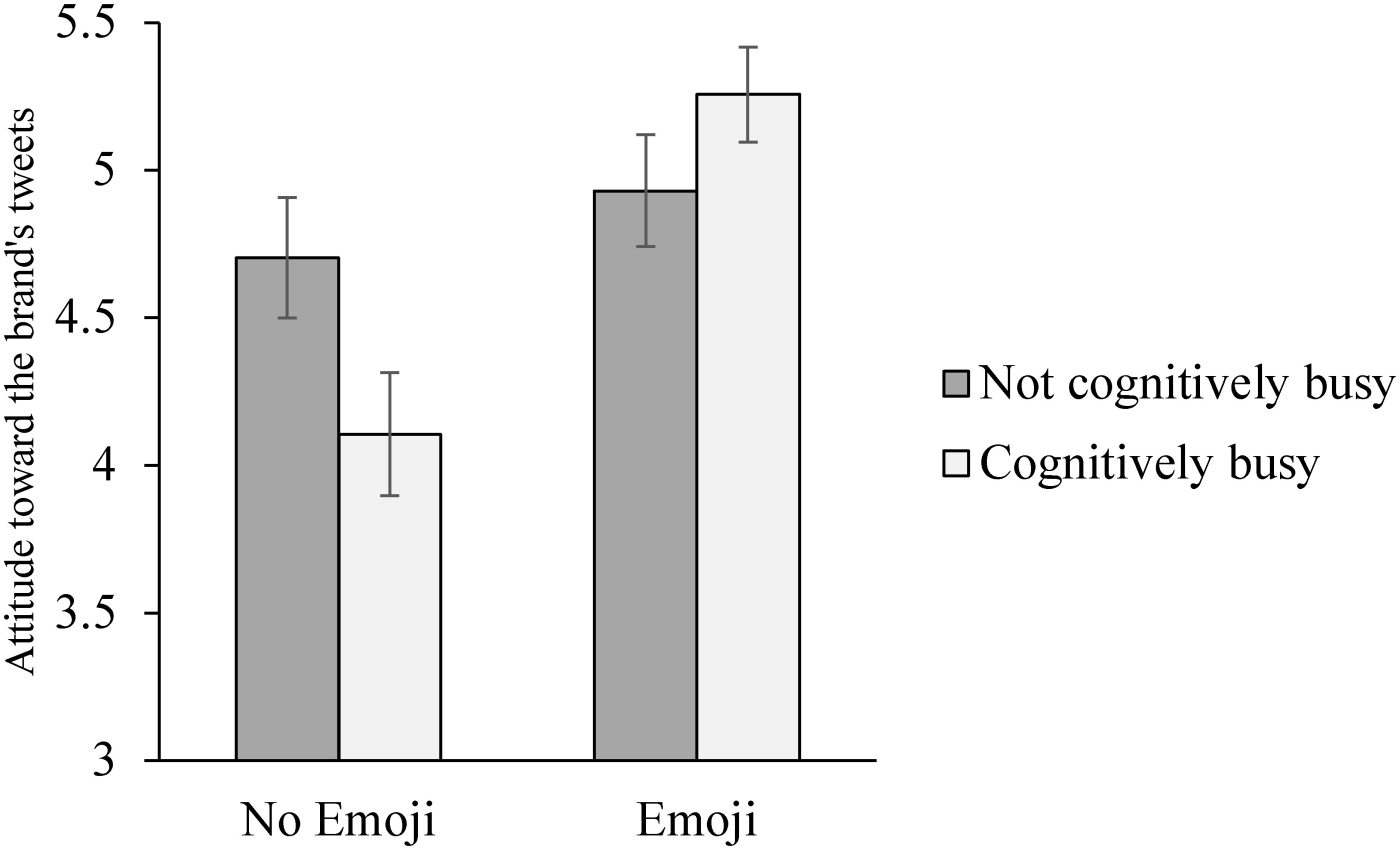
Results of Study 1 (error bars: ±1 SE).

The difference in attitude toward the brand’s social media activity was analyzed in a 2 (cognitive busyness: not busy vs. busy) × 2 (emoji: yes vs. no) between-subjects ANOVA (see Figure 1). The results revealed a significant main effect of emoji [*F* (1, 356) = 5.83, *p* < 0.05, η^2^ = 0.02] and an insignificant main effect of cognitive busyness [*F* (1, 356) = 0.51, *p* = 0.48, η^2^ = 0.001]. More importantly, we found a significant cognitive busyness by emoji interaction, *F* (1, 356) = 13.02, *p* < 0.01, η^2^ = 0.04. Planned contrast analyses further revealed a significant simple effect of emoji use under conditions of cognitive busyness, such that participants reported more favorable attitudes in the emoji condition (*M* = 5.26, SD = 1.54) than in the no-emoji condition (*M* = 4.11, SD = 1.92), *t* (175) = 4.29, *p* < 0.001, *d* = 0.65, 95% CI [0.62, 1.68]. In contrast, under conditions of low cognitive busyness, the effect of emoji use was not significant, *p* = 0.41.

### 3.3 Discussion

The results of Study 1 provide initial support for our hypothesis, suggesting that when people are cognitively busy, emojis play a critical role in shaping evaluative judgments. We speculate that, in the absence of cognitive load, people engage in more reflective processing; however, cognitive busyness limits their capacity to decode nuanced message content, leading to greater reliance on heuristic processing (i.e., a low-effort cognitive style). In this state, emojis indeed serve not only as visually salient cues but also as rapid affective shortcuts, such that positive emotions elicited by emojis are used as a proxy for attitudes toward brand social media content.

Yet, it remains still unclear how emojis enhance message effectiveness through these affective shortcuts. Drawing on the Affect Infusion Model (Forgas, 1995), emojis can be understood as salient paralinguistic cues that efficiently convey affect and thereby facilitate affect-based evaluations during social media browsing. In parallel, the Persuasion Knowledge Model (Friestad and Wright, 1994) suggests that consumers typically activate persuasion knowledge to identify and cope with persuasive intent. However, we believe cognitive busyness can reduce the likelihood that such knowledge is activated and applied. Together, these perspectives suggest that cognitively busy environments amplify affect infusion by not only constraining controlled processing but also weakening persuasion knowledge activation, thereby increasing reliance on readily accessible affective cues such as emojis. To address why emojis produce different downstream consequences across levels of cognitive busyness, Study 2 examines a potential underlying mechanism driving these effects.

## 4 Study 2

Study 2 aims to further examine why paralinguistic visuals that convey human emotions can either enhance or undermine the effectiveness of brand communications. Using a mediation-based approach, we propose that consumers’ perceptions of selling intent serve as a key underlying mechanism explaining these differential emoji effects. Drawing on prior research (Dresner and Herring, 2010), we argue that emojis primarily perform communicative rather than persuasive functions. For example, nonverbal cues such as gestures or facial expressions (e.g., a wink when making a promise) often facilitate effective message delivery. Similarly, emojis may help brands convey intended speech acts (e.g., a winking face accompanying a promise).

However, when consumers are not cognitively busy and are therefore more reflective–able to allocate greater cognitive resources and attend closely to message content–these communicative cues may produce the opposite effect by activating persuasion knowledge. Under such conditions, emojis may heighten perceptions of persuasive (or selling) intent, reducing their effectiveness. In contrast, when consumers are cognitively busy and engaged in multiple demanding tasks, their reduced capacity for reflection may lower perceptions of selling intent. Consequently, the disruptive effect of cognitive busyness on reflective processing may, somewhat paradoxically, enhance the effectiveness of emojis in brand communications.

### 4.1 Methods

*A priori* power analysis was conducted using G*Power (α = 0.05, power = 0.80), indicating a required sample size of 102. A total of 125 Amazon Mechanical Turk participants were recruited for Study 2 in exchange for a small monetary incentive. One participant who failed to complete all measures was excluded prior to analysis, resulting in a final sample of 124 participants (48 females; M_age_ = 35.77, SD_age_ = 10.24). All procedures and the cover story were similar to those used in Study 1, except that Study 2 employed a one-factor (cognitive busyness: not busy vs. busy) between-subjects design. We should note that emojis were inherently present for all participants, as they were instructed to watch the American Express commercial “Take Charge,” which features a variety of everyday inanimate objects (e.g., a smiley face created with a coffee cup and saucer, a frowning face formed with a bathtub and shower curtain) arranged to depict emojis.

#### 4.1.1 Cognitive busyness manipulation

Participants in the non-cognitively busy condition were instructed to watch the commercial only, whereas those in the cognitively busy condition were additionally asked to count the spokesperson’s use of personal pronouns during the commercial. A manipulation check item was also included, asking participants to indicate the extent to which they felt they were performing multiple tasks and experiencing reduced attentional resources during the study (single item, 7-point scale ranging from “Not at all” to “Very much”).

#### 4.1.2 Procedure and measures

After the manipulation, participants completed a series of questionnaire items. First, to assess the primary outcome variable, participants reported their evaluative judgment of the commercial, ad liking. Consistent with prior marketing research, ad liking was measured using adapted semantic differential scales that capture consumers’ affective reactions to advertising stimuli (Duff and Faber, 2011; MacKenzie and Lutz, 1989). Specifically, participants responded to two 7-point semantic differential items: dreary–cheery and dislike very much–like very much (Cronbach’s α = 0.71).

Second, we measured perceived selling intent to capture the extent to which participants perceived persuasive or promotional motives underlying the emoji-embedded brand communication. Drawing on the persuasion knowledge literature (Campbell and Kirmani, 2000), participants responded to two items assessing the extent to which they believed the emojis were intended to influence consumers and promote the brand’s offerings (e.g., “To what extent did you feel that the emojis in the ad were intended to encourage you to open a credit card account with American Express?”). Responses were recorded on 7-point scales ranging from “Not at all” to “To a great extent” (Cronbach’s α = 0.82). Higher scores indicated stronger perceptions of selling intent.

In addition to these measures, we assessed participants’ mood to rule out alternative explanations, such as affect-as-information processes (Schwarz and Bless, 1991), which suggest that incidental moods can influence processing style and strategy (Loken, 2006). Mood was measured using six 7-point items adapted from Watson et al. (1988), anchored from “Not at all” to “Very much,” in which participants reported the extent to which they experienced positive (e.g., excited) and negative (e.g., afraid) emotions during the study. Finally, participants completed demographic questions and were debriefed.

### 4.2 Results

#### 4.2.1 Manipulation checks

We conducted an independent-samples *t*-test to verify that participants perceived and experienced the intended differences in cognitive busyness. Results showed that, compared to participants in the non-cognitively busy condition (*M* = 2.11, SD = 1.75), those in the cognitively busy condition reported engaging in more than one task during the study (*M* = 4.06, SD = 2.18), *t* (122) = −5.50, *p* < 0.01, *d* = −0.99, 95% CI [−1.36, −0.61]. Thus, the manipulation was successful.

#### 4.2.2 Mood control

We also ran a test to see whether there was a significant difference in moods between the experimental conditions. The analysis confirmed that there was no significant mood difference between the two groups, *t* (122) = 0.67, *p* = 0.501, *d* = −0.12, 95% CI = [−0.47, 0.23], suggesting that moods did not meaningfully affect the hypothesized effect.

#### 4.2.3 Hypothesis testing

An independent-samples *t*-test revealed a significant effect of cognitive busyness. Specifically, the emoji-embedded advertisement was evaluated more favorably in the cognitively busy condition (*M* = 5.30, SD = 1.03) than in the non-cognitively busy condition (*M* = 4.89, SD = 1.11), *t* (122) = −2.14, *p* < 0.05, *d* = −0.38, 95% CI [−0.74, −0.03]. Also, consistent with our theorizing, participants in the non-cognitively busy condition reported higher levels of perceived selling intent (*M* = 4.93, SD = 1.57) than those in the cognitively busy condition (*M* = 4.19, SD = 1.77), *t* (122) = 2.45, *p* < 0.05, *d* = 0.44, 95% CI [0.08, 0.79]. To further examine the proposed underlying mechanism, we conducted mediation analyses with perceived selling intent as the mediator. Consistent with our predictions, Sobel-Goodman tests indicated a significant indirect effect (ps < 0.05). In addition, a bootstrap analysis based on 5,000 resamples (Preacher and Hayes, 2004) provided further support for the mediation hypothesis. The bias-corrected 95% confidence interval for the indirect effect ranged from −0.42 to −0.06, excluding zero. Thus, the results provide additional evidence that perceived selling intent mediated the effect of cognitive busyness on ad liking.

### 4.3 Discussion

The findings of Study 2 suggest that participants with greater cognitive capacity were more likely to engage in effortful processing and infer persuasive motives underlying the emoji-embedded message. In contrast, cognitively busy participants were less likely to scrutinize the communication and instead relied more heavily on salient affective cues, such as emojis. As a result, the emoji-embedded advertisement generated more favorable evaluations among cognitively busy participants than among their non-cognitively busy counterparts.

This implies that emojis function as communicative cues in brand messages. When brands incorporate emojis, these graphic symbols can enhance brand evaluations by increasing ad liking; however, this effect depends on perceived selling intent, which is shaped by individuals’ level of cognitive engagement. Specifically, when individuals are cognitively busy, emojis provide immediate affective information that requires minimal cognitive effort to process, thereby facilitating more favorable evaluations of the brand communication and increasing liking. In contrast, when individuals are not cognitively busy, they are more likely to engage in substantive processing of social media content. In this case, emojis are more critically interpreted as deliberate marketing cues signaling overt persuasion or selling intent, which attenuates positive affect infusion and may even reduce favorable evaluations under more analytical scrutiny.

## 5 General discussion

The present research extends beyond the simple prediction that emojis facilitate communication and enhance brand engagement. Drawing on research on cognitive busyness, multitasking, and the Affect Infusion Model, we identify a key boundary condition that shapes the magnitude of emoji effects and uncover an underlying mechanism explaining how and why these affective shortcuts enhance message effectiveness in social media marketing. Specifically, Study 1 shows that people under cognitive busyness rely more heavily on heuristic processing, using emoji-elicited affect as a shortcut when forming attitudes. Study 2 further demonstrates that emojis can enhance ad liking, but this effect is contingent on perceived selling intent. When cognitive resources are limited, people again rely on immediate affective cues, leading to more favorable evaluations. In contrast, when resources are available, people engage in more deliberative processing, interpret emojis as persuasive signals, and attenuate affect-based responses.

Although we provide robust evidence that emojis can function as rapid affective shortcuts in social media marketing, several methodological limitations should be acknowledged. First, while we employed a well-established dual-task paradigm to manipulate cognitive busyness (i.e., requiring participants to simultaneously complete a primary and secondary task and thereby divide their cognitive resources), future research should incorporate alternative operationalizations (e.g., memory load, time pressure, task switching, or task complexity). Doing so would strengthen the generalizability of our central finding that, under any conditions of elevated cognitive busyness (vs. lower cognitive load), emojis operate as efficient emotional heuristics that increase the likelihood of affect infusion in brand evaluations.

Second, it is important to note that the emojis used in Studies 1 and 2 do not capture the full spectrum of human emotional expression. Specifically, the stimuli consisted primarily of positively valenced emojis, reflecting common brand practice in social media marketing, where positive affective cues are preferred. As a result, our understanding remains limited regarding how consumers respond to marketing messages incorporating emojis that vary in emotional valence (e.g., positive vs. negative) and intensity (e.g., high vs. low arousal).

Also, one potential concern is that emojis were presented across different media formats: static social media posts in Study 1 and an animated television commercial in Study 2, which could introduce a confounding factor. From a form-factor perspective, the medium through which emojis are experienced may influence the intensity of consumer responses. Prior research suggests that animated stimuli generally attract greater attention and evoke stronger arousal than comparable static stimuli because motion enhances perceptual vividness and emotional engagement (Sundar and Kalyanaraman, 2004).

Nevertheless, we believe that the fundamental symbolic meanings and affective associations conveyed by the emojis in Studies 1 and 2 remain relatively stable across these contexts. Dual-coding and related information-processing perspectives suggest that consumers can derive affective meaning from visual symbols regardless of the presentation format, although dynamic audiovisual content may amplify the strength of these responses through greater sensory stimulation and attentional engagement (Simmonds et al., 2020). Accordingly, emojis embedded in static social media posts and those appearing in animated advertising content are likely to generate qualitatively similar affective and evaluative responses, even if the magnitude of those responses differs. Specifically, animated contexts may elicit stronger reactions due to their enhanced vividness and arousal potential. Future research could directly examine this possibility by comparing the persuasive effects of emojis across different media formats.

Third, given the growing use of emojis as AI-optimized micro-persuasion tools, an important avenue for future research lies in leveraging artificial intelligence (AI) to generate, recommend, and optimize emoji usage in marketing contexts. AI-driven systems can reduce reliance on managerial intuition by identifying emotionally congruent emojis through sentiment analysis and generative modeling, predicting engagement outcomes, and selecting emoji configurations that maximize affective resonance (see Hotz-Behofsits et al., 2025). Although the present research draws on emojis produced and suggested by established scholarly sources (Rodrigues et al., 2018), future work should consider more bespoke, brand-aligned emoji designs to examine whether more nuanced or brand identity-consistent affective cues differentially shape consumer evaluations under conditions of cognitive busyness.

Fourth, future research could enhance our understanding of emoji-driven affective responses by combining traditional self-report and experimental approaches with more sophisticated neuroscientific and psychophysiological techniques. Although self-report measures effectively capture perceived affective experiences, physiological measures such as electroencephalography (EEG), facial electromyography (EMG), and electrodermal activity (EDA) can provide insight into spontaneous emotional responses that participants may not be able to accurately articulate.

For example, EEG can help identify the temporal dynamics of participants’ neural responses to marketers’ emoji use, suggesting how quickly emotional information is processed and integrated into judgment formation. Also, facial EMG and EDA can capture subtle affective reactions and physiological arousal elicited by emojis, offering indicators of emotional valence and intensity that complement self-reported evaluations (Cacioppo et al., 2007; Venkatraman et al., 2015). Such measures may be especially valuable for uncovering affective mechanisms that operate automatically (or outside conscious awareness), thereby providing deeper insight into how emojis influence consumer perceptions of brands and marketing communications.

Fifth, although MTurk has become a widely used platform for behavioral and consumer research, researchers have identified several limitations associated with its use. These concerns include reduced experimental control, inattentive responding, and participants’ prior exposure to common research paradigms. More recent research has raised additional concerns regarding the quality and representativeness of MTurk data. For example, some studies suggest that MTurk samples may increasingly include inattentive or low-effort respondents, as well as non-human participants such as automated bots (Douglas et al., 2023). Other scholars have argued that MTurk workers are not representative of any clearly defined general population and have highlighted potential self-selection biases stemming from the prevalence of experienced or “professional” survey participants, as well as demographic imbalances within the platform (Kennedy et al., 2020). Accordingly, future research could strengthen the generalizability of the present findings by utilizing alternative participant recruitment platforms (e.g., Prolific), drawing from more diverse participant pools, and replicating the study across different sampling frames and data collection methods.

Lastly, although our sample size was determined *a priori* to ensure adequate statistical power for detecting the focal experimental effect, mediation analyses typically require larger samples because indirect effects are often smaller in magnitude than total effects (Hayes, 2017). Accordingly, the mediation finding in Study 2 should be interpreted with appropriate caution and would benefit from replication in larger samples to further establish its robustness.

## 6 Conclusion

Overall, this research advances understanding of emoji use in marketing communications. Drawing on cognitive busyness perspectives and the Affect Infusion Model, we show that emojis function as affective paralinguistic cues whose persuasive impact varies systematically as a function of individuals’ cognitive resources and their interpretations of persuasive intent. We hope that our findings and theoretical contributions contribute to both marketing research and practice by providing a more nuanced understanding of emojis as context-dependent yet strategically significant elements of marketing communication.

## Data Availability

All materials and data used in this research will be made available by the author upon request.

## Footnotes

1 All materials used in this research and data are available from the corresponding author upon request.
